# FRET-Based Semiconducting Polymer Dots for pH Sensing

**DOI:** 10.3390/s19061455

**Published:** 2019-03-25

**Authors:** Jiemei Ou, Huijun Tan, Zhong Chen, Xudong Chen

**Affiliations:** 1 Key Laboratory for Polymeric Composite and Functional Materials of Ministry of Education, Guangdong Engineering Technology Research Center for High-Performance Organic and Polymer Photoelectric Functional Films, School of Chemistry, Sun Yat-sen University, Guangzhou 510275, China; oujiemei12@163.com (J.O.); tanhj5@mail2.sysu.edu.cn (H.T.); 2 National Engineering Research Center for Healthcare Devices, Guangdong Key Lab of Medical Electronic Instruments and Polymer Material Products, Guangdong Institute of Medical Instruments, Guangzhou 510500, China

**Keywords:** semiconducting polymers, polymer dots, FRET, pH sensing

## Abstract

Förster resonance energy transfer (FRET)-based polymer dots (Pdots), fabricated by semiconducting polymers and exhibiting excellent properties, have attracted much interest in the last decade, however, full polymer-dot-based pH sensors are seldom systematically exploited by researchers. In this work, we constructed a kind of blend polymer dot, utilizing poly[(9,9-dihexyl-9H-fluorene-2,7-vinylene)-co-(1-methoxy-4-(2-ethylhexyloxy)-2,5-phenylenevinylene)] (PFV) as the donor, poly[2,5-bis(3′,7′-dimethyloctyloxy)-1,4-phenylenevinylene] (BDMO-PPV) as the acceptor, and polysytrene graft EO functionalized with carboxy (PS-PEG-COOH) to generate surface carboxyl groups. This type of Pdot, based on the FRET process, was quite sensitive to pH value changes, especially low pH environments. When the pH value decreases down to 2 or 1, the fluorescence spectrum of Pdots-20% exhibit spectral and intensity changes at the same time, and fluorescence lifetime changes as well, which enables pH sensing applications. The sharpening of the emission peak at ~524 nm, along with the weakening and blue shifts of the emission band at ~573 nm, imply that the efficiency of the energy transfer between PFV and BDMO-PPV inside the Pdots-20% decreased due to polymer chain conformational changes. The time-resolved fluorescence measurements supported this suggestion. Pdots constructed by this strategy have great potential in many applications, such as industrial wastewater detection, in vitro and intracellular pH measurement, and DNA amplification and detection.

## 1. Introduction

Förster resonance energy transfer (FRET) is an non-radiative energy transfer mechanism between two chromophores, based on a well-defined distance-dependent dipole–dipole coupling within a donor–acceptor pair [1], discovered by the German scientist, Theodor Förster [2]. The efficiency of this energy transfer is inversely proportional to the sixth power of the distance between the donor and acceptor, which is a useful tool to measure a small distance change due to its extremely high sensitivity. Organic fluorophores including fluorescein, rhodamine, coumarin, Alexa Fluor and cyanine are the most frequently used FRET materials owing to their narrow emission, easily purchased commercial products, and readiness for chemical modification. Early in 2002, the Yi Lu group [3] used a trifluorophore-labeled “8–17” DNAzyme for studying complicated biomolecular structure and dynamics, and revealed new sophisticated structural changes. Very recently, Selnihhin et al. [4] described the design, construction, and characterization of an optical DNA origami nanobiosensor device using two arrays of donor and acceptor fluorophores to make up a multi-fluorophore FRET pair that results in a high-output signal for microscopic detection of single devices. In addition, multiplexed fluorescent nanocrystals (quantum dots, QDs) with extraordinary optical properties [5,6,7,8], including broad absorption with narrow emission, high photostability, and a large effective Stokes shift that is superior to other common fluorophores, such as FRET donors, acceptors, or even both at the same time, have become the most appropriate candidate in FRET. For example, Freeman et al. [9] demonstrated a strategy for the multiplexed detection of DNA by using different-sized QDs together with exonuclease-catalyzed digestion and target regeneration. Qiu and Hildebrandt [10] developed a multiplexed FRET between a luminescent Tb complex and three different semiconductor QDs to sensitively detect three different miRNA targets in a single sample with very low detection limits. Yet, the poor water solubility and rapid photobleaching of organic fluorophores [11] and inherent toxicity of quantum dots [12] greatly preclude their practical biological applications and make them unfriendly to the environment. Nevertheless, FRET still has great potency in biosensing [13,14,15], bioimaging [16], ratiometric detection [17], immunoassay [18] and pH and ion sensing [19].

In order to tackle the problem, those fluorescent materials need to be optimized or redesigned to enhance the stability and reduce the biotoxicity before application in those fields. Parak’s group [20] prepared nanoparticle-based biosensors that detect the spectral changes of the acceptor at a fixed donor/acceptor distance by introducing the acceptor into the polymer coating, allowing for short acceptor–donor separation, and thus high-energy transfer efficiencies. Chiu’s group [21] developed a new class of nanocomposites, made of polymer dots (Pdots) and Qdots with narrow/near infrared (NIR) emission, easy optical tunability, and facile surface chemistry, that exhibit combined functionalities and optical properties that are superior to either type of nanoparticle, and can be utilized for specific in vitro and in vivo cellular imaging. Besides the polymer composites coating or crosslinking with QDs, the pure semiconducting polymer dots, as organic macromolecules, have attracted surging interest because they combine the beneficial signature properties of organic small molecule fluorophores and inorganic QDs, such as signal amplification ability, excellent photostability, and low toxicity, which has been largely exploited in pH and temperature sensing applications [22,23,24,25,26,27] because the pH and temperature are fundamental target parameters broadly used from environmental to biomedical research. However, full polymer-dot-based pH sensors are seldom systematically exploited by researchers. Only Chiu’s group [28] in 2011 previously reported the design and performance of the first pH-sensitive PPE (poly(2,5-di(3′,7′-dimethyloctyl)phenylene-1,4-ethynylene)) Pdots as a platform for designing FRET- based ratiometric pH nanoprobes.

To address the above-mentioned challenges and extend the application of full polymer dots, here we report simple, versatile, and water-soluble pH sensitive multiple polymer dots blended with poly[(9,9-dihexyl-9H-fluorene-2,7-vinylene)-co-(1-methoxy-4-(2-ethylhexyloxy)-2,5-phenylenevinylene)] (PFV), poly[2,5-bis(3′,7′-dimethyloctyloxy)-1,4-phenylenevinylene] (BDMO-PPV) and polysytrene graft EO functionalized with carboxy (PS-PEG-COOH), in which the PS-PEG-COOH acts as a hydrotrope that improves the hydrophilia of polymer nanoparticles, enabling further bioconjugation for biological applications. This kind of Pdots, based on the FRET process, was sensitive to pH value changes, exhibiting great potential for pH sensor design.

## 2. Materials and Methods

### 2.1. Materials

PFV (95:5 mole ratio), BDMO-PPV, HPLC grade tetrahydrofuran (THF) and (3-Aminopropyl)- triethoxysilane (APTES) were purchased from Sigma-Aldrich (Milwankee, USA). PS-PEG-COOH was purchased from Polymer Source Inc. Ltd., (Dorval, QC, Canada; Back bone PS Mn = 6500; each branch Mn = 4600; Mn total = 30 000, Mw/Mn = 1.3). Hydrochloric acid (AR, 36.5%) and sodium hydroxide (AR) were ordered from Guangzhou Chemical Reagent Factory (Guangzhou, China). Ultra-pure water used for all the experiments was prepared with a Milli-Q water system (Millipore, Bedford, MA, USA).

### 2.2. Preparations of Pdots

Suspensions of Pdots were prepared by nanoprecipitation from a solvent mixture containing equal amounts of PFV and PS-PEG-COOH. Briefly, PFV, BDMO-PPV, PS-PEG-COOH were dissolved in THF to make stock solutions. The aforehand solution mixtures with constant PFV and PS-PEG-COOH concentrations were prepared by mixing 0.5 mL PFV solution (0.22 mg/mL) and 22 μL PS-PEG-COOH solutions (1.0 mg/mL). Varying amounts of BDMO-PPV solution were then mixed with the mentioned solution mixtures to produce the final solution mixtures with BDMO-PPV/polymer fractions ranging from 0 to 20 wt%. To eliminate errors from the volume differences, additional THF was added to make volumes of the final solution mixtures to be 1 mL. In a typical preparation, a 1 mL quantity of the final solution mixtures was injected quickly into 10 mL of ultrapure water under ultrasonication. After another 15 min sonication, the mixtures were moved to a dark environment for 48 h for THF evaporation. The resulted suspensions were filtered through a 0.2 μm micro filter before being used for measurements.

### 2.3. Methods

The Pdots were evaluated by a JEM-2010HR transmission electron microscope (TEM, Japan Electronics Corporation, Tokyo, Japan) and a Dimension Fastscan atom force microscope (AFM) instrument (Bruker, Germany). The AFM measurements were taken on the PeakForce QNM tapping mode. Samples for AFM measurements were absorbed on the glass substrate with an APTES modification.

UV-visible absorption spectra were recorded with a PerkinElmer Lamda750 UV-vis-NIR spectrophotometer (PerkinElmer, Waltham, MA, USA. Fluorescence measurements were performed on a FLS-920 spectrometer (Edinburgh Instruments Ltd., Livingston, UK). Fluorescence spectra were acquired under the excitation by a Xe lamp at 400 nm. For fluorescence lifetime measurements, a 405 nm picosecond pulse laser was used to excite the samples. The instrument response function (IRF) was recorded on a 30% colloidal silica sample. All decay traces were fit by a procedure involving reconvolution of a multiexponetial function $F\left(t\right)=A+\sum_{i=1}^{n}B_{i}\exp\left(-t/\tau_{i}\right)$ with the IRF, where *A* is a constant, $\tau_{i}$ are the decay times, and $B_{i}$ are the amplitudes of the components at *t* = 0, and n is the number of decay time. Considering the weak absorbance of the Pdots, HCl or NaOH aqueous solutions with concentrations of 10 M or 5 M were added into 2 mL of the Pdot suspensions to modulate the pH value from 1 to 13 for the absorption spectra measurements. For fluorescence measurements, 100 μL of the prepared suspensions of Pdots was diluted into 2 mL HCl or NaOH aqueous solutions with various pH values. All of the above mixtures were shaken for 1 min before being measured.

## 3. Results and Discussion

**Figure 1:**
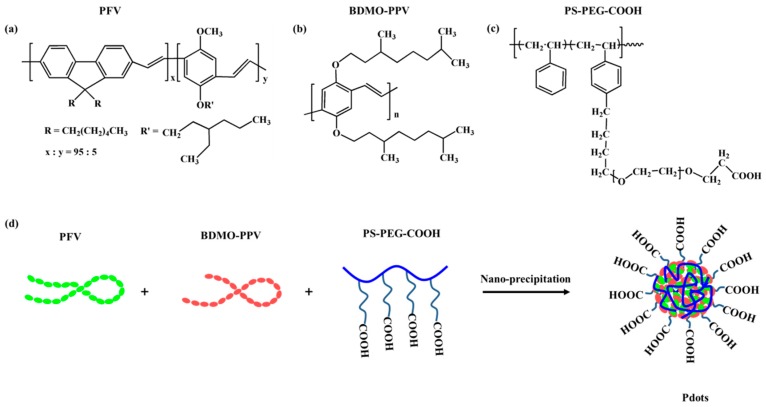
Chemical structures of the utilized polymers (**a**–**c**) and schematic illustration of the nanoprecipitation procedure for the Pdots preparation (**d**).

The Pdots were prepared from the three polymers (PFV, BDMO-PPV, PS-PEG-COOH) by using the nanoprecipitation method. The preparation involves a rapid mixing of a dilute solution of polymers dissolved in THF with water, which leads to a sudden decrease in solvent quality, resulting in the formation of a suspension of hydrophobic polymer nanoparticles. Figure 1 presents the chemical structures of PFV, BDMO-PPV, PS-PEG-COOH and the formation procedure of the blend Pdots. The blend Pdots consist of the visible-light-harvesting polymer PFV as the donor and the orange emitting polymer BDMO-PPV as the acceptor. The amphiphilic polymer, PS-PEG-COOH, was used to generate surface carboxyl groups (Figure 1d), which makes the suspensions clear and stable for weeks without signs of aggregation, and enables further surface conjugation for biological applications.

**Figure 2:**
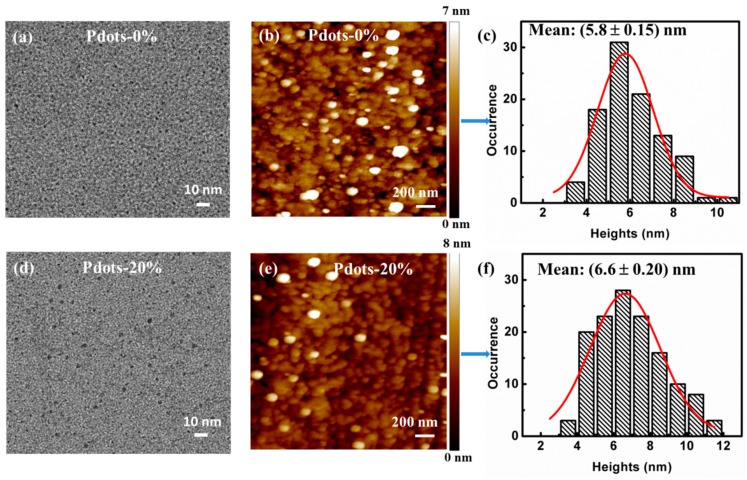
TEM image, AFM topography and the corresponding histogram of height of Pdots doped with different BDMO-PPV contents: (**a**–**c**) Pdots- 0%; (**d**–**f**) Pdots-20%.

TEM images indicate that both the functionalized Pdots with 0% and 20% BDMO-PPV dopant have small particle diameters, less than 10 nm (Figure 2a,d). As the small sizes and low contrast grades of the Pdots inhibit obtaining clear enlarged TEM images, the size distributions of Pdots were further verified by particle height analysis of representative AFM images. As show in Figure 2b,e, the Pdots doped with both 0% and 20% BDMO-PPV contents (Pdots-0% and Pdots-20%) exhibit spherical morphology (white dots), as expected for glassy polymers in this size range, consistent with surface free energy considerations [29]. Note that the observed particle sizes are much larger than the real sizes because of the stretching of the AFM probe. Hence, the 3D morphologic images of Pdots-0% and Pdots-20% display particle sizes closer to the real sizes (shown in Figure S1). A particle size distribution of 5.8 ± 0.15 nm was determined by the AFM for the Pdots-0% (Figure 2c), while it was 6.6 ± 0.20 nm for the Pdots-20% (Figure 2f), indicating that the sizes of the BDMO-PPV doped Pdots were only minimally affected by the doping concentrations. The lateral dimensions from the AFM image are somewhat larger than the height due to the radius of curvature of the AFM tip [30].

**Figure 3:**
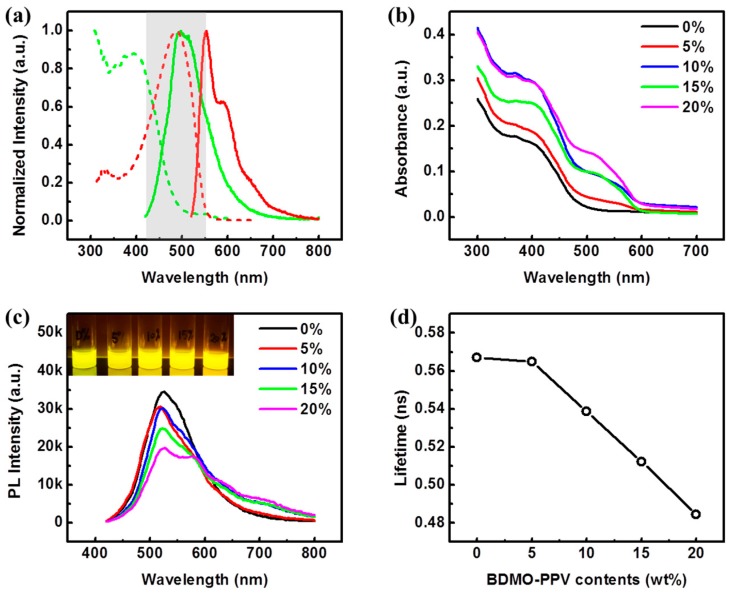
(**a**) Normalized absorption (dash line) and fluorescence (solid line) spectra of PFV (green) and BDMO-PPV (red) solutions; (**b**) Absorption spectra of Pdots doped with different BDMO-PPV contents; (**c**) Fluorescence spectra of Pdots doped with different BDMO-PPV contents (The inset photo shows the corresponding colors under a 365 nm UV lamp); (**d**) Average lifetimes of Pdots doped with various BDMO-PPV contents.

Figure 3a shows the UV-vis absorption spectra and fluorescence spectra of PFV and BDMO-PPV in THF solutions. As marked with the light grey, the absorption of BDMO-PPV possesses good overlap with the fluorescence spectrum of PFV in the ~420–550 nm range, cohering with the requirement for efficient energy transfer via the Förster mechanism. The absorption spectra of Pdots doped with different BDMO-PPV contents are displayed in Figure 3b. Two absorption bands at ~400 nm and 510 nm were observed in spectra of the blend Pdots in high dopant contents. The ratio of the two absorption bands varied with the increasing weight fraction of the BDMO-PPV dopant. As shown in Figure 3c, the BDMO-PPV dopant could efficiently quench the PFV emission at relatively low concentrations. Very similar spectral features were observed at moderate doping levels (5%). As the doping concentration increased from 5% to 20%, the intensity of the PFV emission further decreased and the spectral shapes changed, whereas a gradually obvious emission band from BDMO-PPV at ~573 nm was observed. These spectra changes indicate that the energy transfer process from PFV to BDMO-PPV take places inside the Pdots. An energy transfer efficiency of 43% was observed at 20% doping, where the energy transfer efficiency is given by *η*_ET_ = 1 − *I*/*I*_0_, and *I*, *I*_0_ is the donor (PFV) fluorescence intensity in presence or absence of acceptor (BDMO-PPV). The occurrence of energy transfer could be verified by the decreased average lifetimes of PFV with increasing BDMO-PPV dopant as well, shown in Figure 3d. The decay trace of PFV doped with various BDMO-PPV contents was adequately fit by a biexponential (Figure S2). These average lifetimes obtained from the fitting results are consistent with the physical picture of exciton diffusion energy transfer [29].

**Figure 4:**
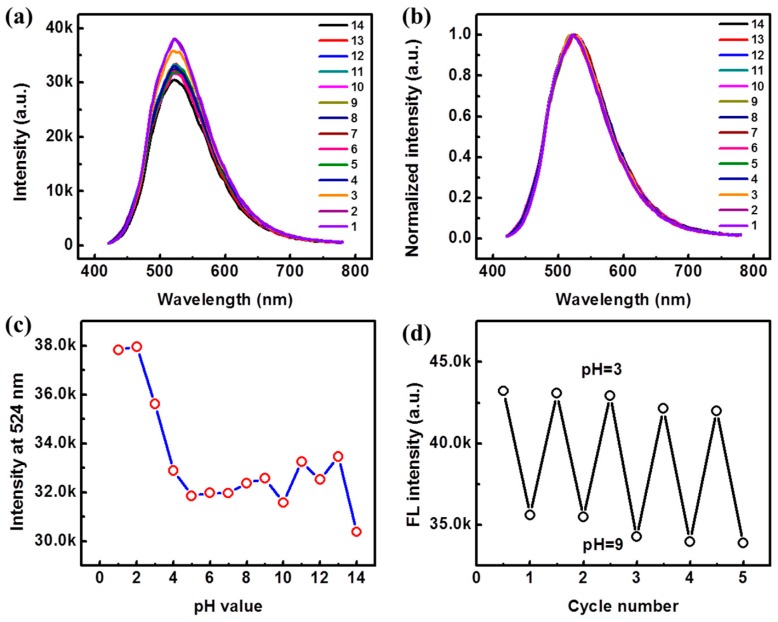
Fluorescence properties of Pdots-0% at various pH values: (**a**) Fluorescence spectra; (**b**) Normalized fluorescence spectra; (**c**) The evolutions of the maximum emission peak at 524 nm with various pH values; (**d**) Fluorescence intensity at 524 nm upon repeatedly switching pH between 3 and 9.

As the Pdots were blended with the conjugated polymers, PFV and BDMO-PPV, and functionalized with -COOH on the surfaces, it could be inferred that the fluorescence of the blend Pdots would response to pH changes. Firstly, the Pdots undoped with BDMO-PPV (Pdots-0%) were investigated. Figure S3a shows the absorption spectra of the Pdots-0% (undoped) in hydrochloric acid/sodium hydroxide aqueous solutions with different pHs, where weak absorption was observed at about 400 nm. As the pH value decreased from 7 to 4, the absorption spectra of the Pdots-0% were feckly unchanged. When the pH value decreased further, the absorption of Pdots-0% increased, along with the baselines of the absorption spectra, due to the strong scattering of the nanoparticles [31]. Under basic conditions (pH = 8–13), the absorption spectra of Pdots-0% changed the absorbance with irregularity. The fluorescence spectra of Pdots-0% in acid or basic solution exhibit similar shapes to the spectrum at a neutral environment (pH = 7) but have different intensities with changing pH values, as illustrated in Figure 4a,b. Figure 4c shows the evolution of the intensities of the emission peak at 524 nm at various pH values, in which a trend of increasing intensity with decreasing pH from 7–1 can be observed, while no regularity can be seen in higher pH levels. Regardless, the fluorescence intensity of the Pdots-0% can exhibit a progressive recovery between pH 3 and pH 9, as shown in Figure 4d. Very slight blue shifts can be also observed for the fluorescence spectra of the Pdots-0% under different pH values compared with that at pH = 7. However, these slight blue shifts and the single emission peak only enable an estimation of response to pH changes through the intensity changes, which may not be accurate enough in many cases because the fluorescence intensity is affected by many factors.

**Figure 5:**
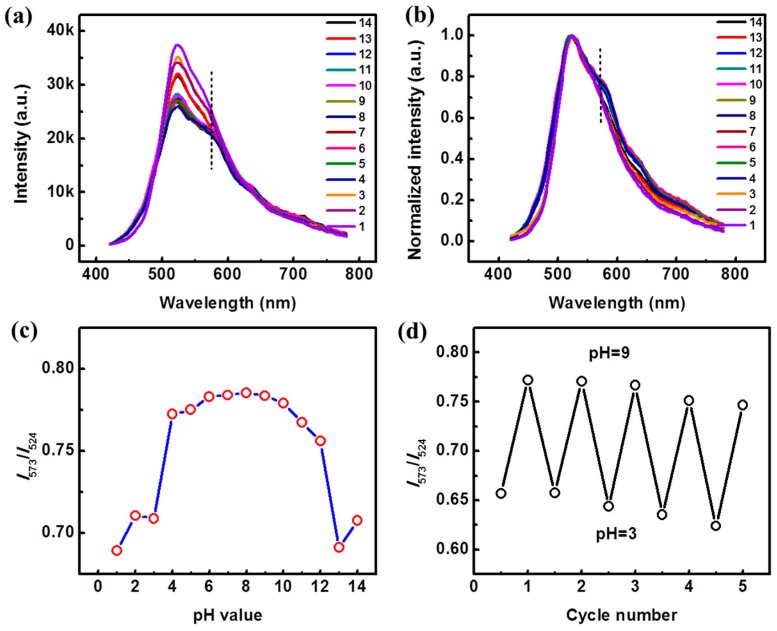
Fluorescence properties of Pdots-20% at various pH: (**a**) Fluorescence spectra; (**b**) Normalized fluorescence spectra; (**c**) Plot of the relative fluorescence intensity ratios (*I*_573_/*I*_524_) in response to pH values; (**d**) The intensity ratios (*I*_573_/*I*_524_) upon repeatedly switching pH between 3 and 9.

In consideration of that, ratiometric measurement is a better solution for pH sensing. As there are two emission peaks for the Pdots doped with 20% BDMO-PPV contents (Pdots-20%), more possibilities can be obtained. The absorption spectra of Pdots-20% at various pH values are displayed in Figure S3b. Compared with the absorption spectra of Pdots-0%, a new absorption band at ~ 510 nm is observed beside the absorption band of PFV at ~400 nm, which contributes to BDMO-PPV dopants. Similar to those for Pdots-0%, the baselines increase intensely when the pH values decrease down to 2 or 1. More interestingly, the absorption band at about 510 nm also weakens as the pH values decrease down to 2 or 1, indicating polymer chain conformational changes inside the Pdots. The trends in the fluorescence spectra of Pdots-20% with changing pH values are different from the spectra of Pdots-0%, as shown in Figure 5a,b. The fluorescence spectra of Pdots-20% exhibit almost the same shapes and intensities. However, a dramatic increase of intensity, along with a spectral shape change, is observed under extremely low (1–3) or high (13–14) pH values. The major emission at ~524 nm becomes sharper, while the emission band at ~ 573 nm weakens and blue shifts to 550 nm, becoming a small shoulder peak. Ratiometric detection has been used for sensing in previous reports [32,33], e.g., pH or temperature sensing. The relative fluorescence intensity ratios (*I*_573_/*I*_524_) in response to different pH values are plotted in Figure 5c, from which gradually decreasing trends can be observed as the pH value decreases from 7 to 1 or increases from 7 to 14. Notably, an approximately linear relationship between the intensity ratios and the pH values can be observed in the range of 9–12. More interestingly, mutations can be also observed when the pH decreases from 4 to 3 or increases from 12 to 13. These features for Pdots-20% may be attributed to the decreased efficiency of the energy transfer process due to the polymer chain conformational changes or the degrading of the BDMO-PPV under harsh conditions. To get insight into that, a reference system comprising PFV as the donor and BDMO-PPV as the acceptor, and adding PS as a matrix, was measured under the same pH levels. Fluorescence spectra of PS-Pdots-0% and PS-Pdots-20% under various pH values are shown in Figure S4, which shows that the PS-Pdots-0% do not exhibit any irregularity changes of the intensity with various pH values, while the PS-Pdots-20% exhibit two mutations of the intensities under pH ranges from 3–1 and 12–14. These results indicate that the Pdots comprising with PS matrix are not as stable as those comprising with PS-PEG-COOH. Figure 5d shows the reversibility of Pdots-20% for cyclic detection at pH 3 and 9, which implies that there is no degradation of components of the Pdots under acid or basic conditions.

The mechanism of sensing for Pdots-20% can be explained by the electrostatic effects, including a conformational change of the polymers to minimize electrostatic repulsion. There is an ionization equilibrium in the Pdots-20% suspensions (just like adding them into neutral conditions, pH = 7). When the Pdots-20% were added into a NaOH solution, the carboxylic acid groups on their surfaces react with the OH^−^. Taking account of the molecular weight of PS-PEG-COOH (about 5 carboxyl groups in each PS-PEG-COOH molecule), almost all carboxyl groups are neutralized by the OH^−^ when 100 μL of Pdots-20% are mixed with 2 mL NaOH aqueous solution with pH = 8, resulting in a carboxylate salts-like solution. In this solution, the PS-PEG-COO^−^ chains in the Pdots-20% are in a relatively extended conformation due to the repulsive negative charges from the end group (-COO^−^), enabling effective energy transfer between PFV and BDMO-PPV. At a higher pH, the introduction of Na^+^ decreased the degree of dissociation due to the common-ion effect, leading to a more curled conformation of PS-PEG-COOH, thus decreasing the efficiency of the energy transfer between PFV and BDMO-PPV. The decreased values of *I*_573_/_524_ seen with increasing pH from 8 to 14 supports this mechanism. In addition, the extended polymer chains at high pH could further expose the Pdots-20% to the aqueous solution. After acidifying, the additional H^+^ promotes the protonation of the carboxylic acid groups and leads to a decrease in interchain electrostatic repulsion, resulting in more curled conformations of PS-PEG-COOH chains as well. The further curled chain conformations may inhibit the energy transfer between PFV and BDMO-PPV, which correspond to the decreasing value of *I*_573_/_524_ in the pH range from 7 to 1. The two mutations observed at pH 3 or pH 13 may correlate with a charge transfer excited state of BDMO-PPV. The decreased fluorescence of BDMO-PPV at high pH is also constant with a charge transfer excited state that increases its nonradiative decay rate [34].

Moreover, the time-resolved fluorescence spectra of Pdots-20% at various pHs were measured upon excitation at 405 nm and collection at 524 nm (exciting and collecting at PFV), as seen in Figure S5. All decay traces were fitted with biexponential functions and the results are summarized in Table 1. There were no significant differences in average lifetimes ($\tau_{avg}$) extracted from the decay traces for Pdots-20% at pH ranges from 7–4. However, as the pH value decreased to 2 or 1, the proportion of the fast component (*B*1) increased conspicuously. The progressive increase of the fast component for Pdots-20% at low pH values (1 or 2) is consistent with emission spectral observations, supporting the suggestion that the efficiency of the energy transfer inside the Pdot-20% decreases as the polymer chain conformations change at low pH environments. At high pH levels, the average lifetimes of Pdots-20% are longer than those at acid conditions and decrease with increasing pH values. The decreased lifetimes and increased fast component for Pdots-20% at pH 13 and 14 also indicate decreased efficiency of the energy transfer inside the Pdots-20%.

As mentioned above, the Pdots-20% sensitively sense the low pH environment; the spectral shape, intensity and fluorescence lifetime are response to low pH values. Furthermore, the Pdots constructed from blend polymers are eco-friendly with bright emission, excellent photostability, and low toxicity to biological applications. Although the pH sensing results in the pH range of 4–12 presented here seem to not be so desirable, some improvement can manipulate the properties of these type blend Pdots to sensitively respond to the pH value well over a large range. Another study of Pdots-2% (PFV doped with 2% BDMO-PPV) showed rather encouraging results for pH sensing (Figure S6). Therefore, it can be predicted that Pdots constructed by this strategy have great potential for use in industrial wastewater detection, in vitro and intracellular pH measurement, DNA amplification and detection, and so on.

## 4. Conclusions

The FRET-based Pdots fabricated by semiconducting polymers exhibit excellent properties, including signal amplification ability, excellent photostability, and low toxicity, attracting much interest over the last decade. In this work, we constructed a kind of blend polymer dot that utilizes PFV as the donor, BDMO-PPV as the acceptor, and PS-PEG-COOH to generate surface carboxyl groups. This type of Pdot, based on the FRET process, was quite sensitive to pH value changes, especially low pH. When the pH value decreased down to 2 or 1, the fluorescence spectrum of Pdots-20% exhibited spectral and intensity changes at the same time, and the fluorescence lifetime changed as well, which enables pH sensing applications. The sharpening of the emission peak at ~524 nm along with the weakening and blue shifts of the emission band at ~ 573 nm implies that the efficiency of the energy transfer between PFV and BDMO-PPV inside the Pdots-20% decreased due to the polymer chain conformational changes. The time-resolved fluorescence measurements supported this suggestion. The pH sensing result presented here is not so desirable in a range of 3–7 and much more effort is needed to investigate the FRET-based blend Pdots for pH sensing using pure polymers. Nevertheless, the strategy applied here introduces a new possibility for generating novel nanoprobes for pH sensing, which can be used in many applications, such as industrial wastewater detection, in vitro and intracellular pH measurement, and DNA amplification and detection.

## Figures and Tables

**Table 1:** Summary of the fitting results of the decay traces for Pdots-20% at various pH values.

pH	*τ*_1_ (ns)	*τ*_2_ (ns)	*B*_1_	*B*_2_	*τ*_avg._ (ns)
1	0.35	1.14	0.84	0.16	0.47
2	0.34	1.07	0.82	0.18	0.48
3	0.33	1.17	0.77	0.23	0.51
4	0.32	1.13	0.74	0.26	0.52
5	0.34	1.16	0.76	0.24	0.53
6	0.31	1.10	0.73	0.27	0.52
7	0.32	1.11	0.74	0.26	0.53
8	0.45	1.27	0.73	0.27	0.67
9	0.42	1.24	0.72	0.28	0.65
10	0.43	1.21	0.76	0.24	0.62
11	0.44	1.22	0.75	0.25	0.63
12	0.46	1.30	0.78	0.22	0.64
13	0.42	1.14	0.81	0.19	0.56
14	0.40	1.22	0.81	0.19	0.56

Note: *τ*_avg_ represents the average lifetime, *τ*_avg_ = *τ*_1_ × *B*_1_ + *τ*_2_ × *B*_2_.

## Supplementary Materials

The following are available online at https://www.mdpi.com/1424-8220/19/6/1455/s1, Figure S1: The 3D morphologic images of Pdots-0% and Pdots-20%; Figure S2: Time-resolved fluorescence spectra of Pdots doped with different BDMO-PPV contents; Figure S3: Absorption spectra of Pdots-0% and Pdots-20% at various pH values; Figure S4: Fluorescence spectra of the reference system comprising PFV as the donor and BDMO-PPV as the acceptor and adding PS as a matrix under various pH values; Figure S5: Time-resolved fluorescence spectra of Pdots-20% under various pH values; Figure S6: Fluorescence properties of Pdots-2% at various pH values.
