# Supplementary material for: FRET-Based Semiconducting Polymer Dots for pH Sensing
Source: Sensors (Basel). 2019 Mar 25;19(6):1455. doi: 10.3390/s19061455 (PMC6470647; doi:10.3390/s19061455)
Supplement: Supplementary file 1 [file sensors-19-01455-s001.pdf]

Supplementary Materials

FRET-based semiconducting polymer dots for pH sensing

Jiemei Ou [1,2,†], Huijun Tan [1,†], Zhong Chen [1,*] and Xudong Chen [1,*]

[1] Key Laboratory for Polymeric Composite and Functional Materials of Ministry of Education, Guangdong Engineering Technology Research Center for High-Performance Organic and Polymer Photoelectric Functional Films, School of Chemistry, Sun Yat-sen University, Guangzhou 510275, China; chenzhong@mail.sysu.edu.cn

2 National Engineering Research Center for Healthcare Devices, Guangdong Key Lab of Medical Electronic Instruments and Polymer Material Products, Guangdong Institute of Medical Instruments, Guangzhou, 510500, China; oujiemei12@163.com

* Correspondence: chenzhong@mail.sysu.edu.cn (Z.C.); cescxd@mail.sysu.edu.cn (X.C.); Tel.: +86-189-9838-2617(Z.C.); +86-138-0293-7231(X.C.)

† These authors made equal contributions.

Additional Figures

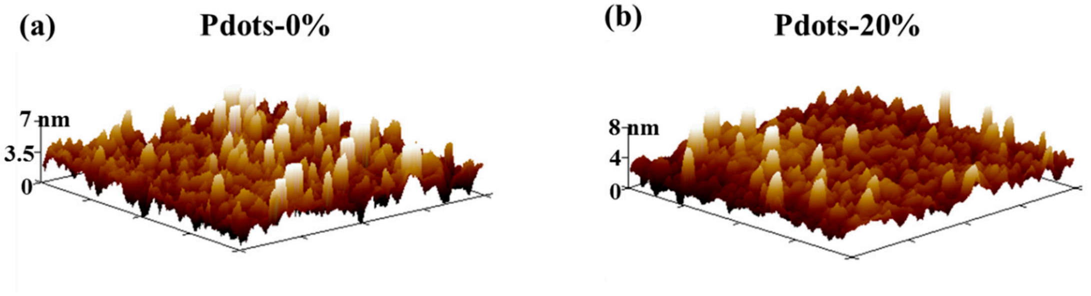

Figure S1. The 3D morphologic images of Pdots-0% (**a**) and Pdots-20% (**b**).

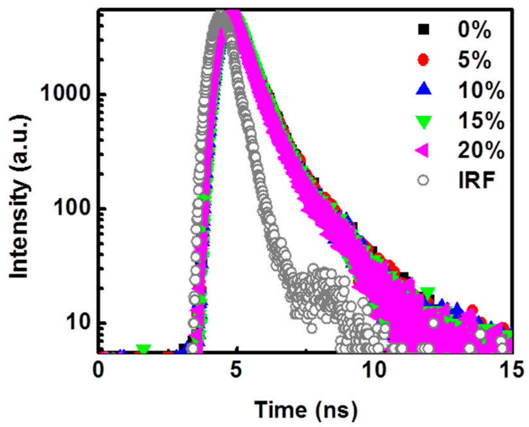

Figure S2. Time-resolved fluorescence spectra of Pdots doped with different BDMO-PPV contents.

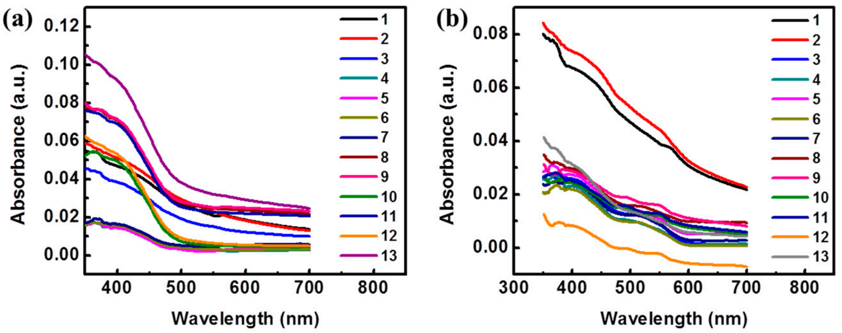

Figure S3. Absorption spectra of Pdots-0% and Pdots-20% at various pH values.

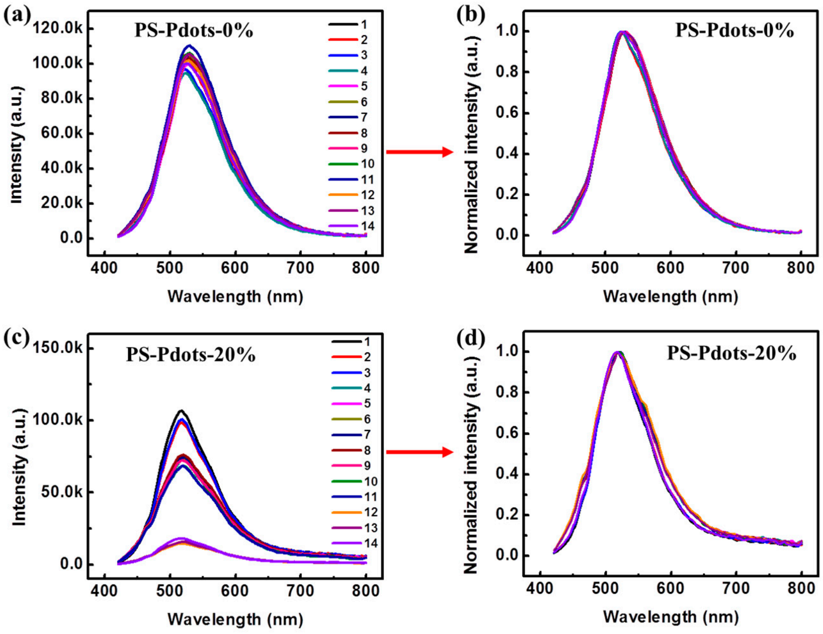

Figure S4. Fluorescence spectra of the reference system comprising PFV as the donor and BDMO-PPV as the acceptor and adding PS as matrix under various pH values: (**a**,**b**) PS-Pdots-0%; (**c**,**d**) PS-Pdots-20%.

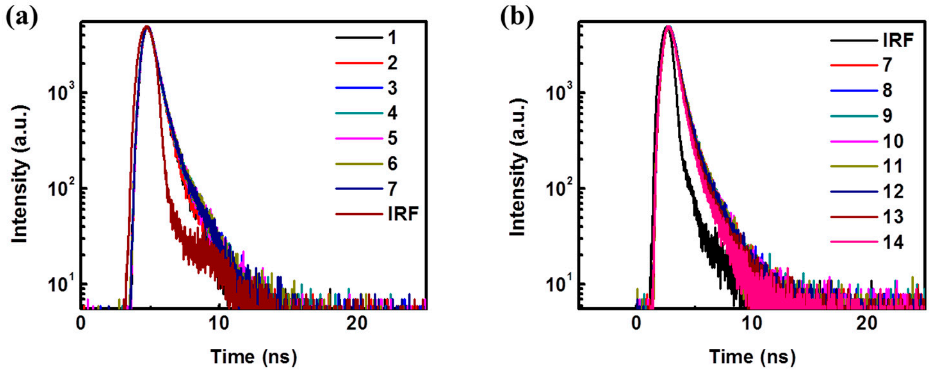

Figure S5. Time-resolved fluorescence spectra of Pdots-20% under various pH values.

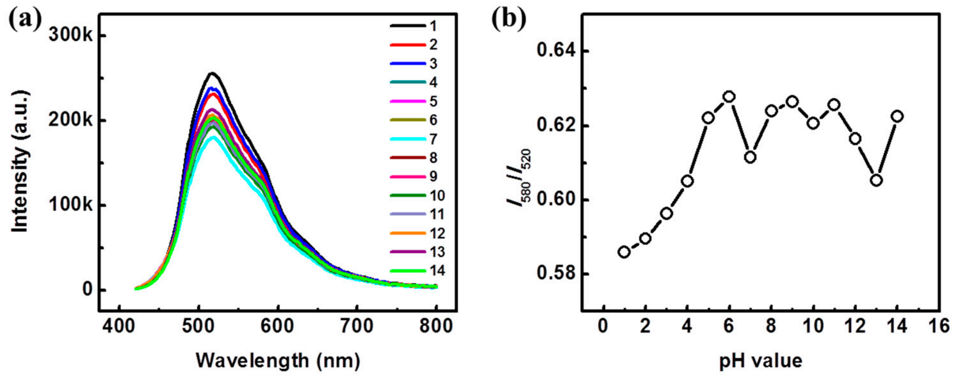

Figure S6. Fluorescence properties of Pdots-2% at various pH: (**a**) Fluorescence spectra; (**b**) Plot of the relative fluorescence intensity ratios (I_{580}/I_{520}) in response to pH values.
